# Transmission Dynamics of HIV-1 Drug Resistance among Treatment-Naïve Individuals in Greece: The Added Value of Molecular Epidemiology to Public Health

**DOI:** 10.3390/genes8110322

**Published:** 2017-11-13

**Authors:** Dimitrios Paraskevis, Evangelia Kostaki, Panagiotis Gargalianos, Georgios Xylomenos, Marios Lazanas, Maria Chini, Athanasios Skoutelis, Vasileios Papastamopoulos, Dimitra Paraskeva, Anastasia Antoniadou, Antonios Papadopoulos, Mina Psichogiou, Georgios L. Daikos, Georgios Chrysos, Vasileios Paparizos, Sofia Kourkounti, Helen Sambatakou, Nikolaos V. Sipsas, Malvina Lada, Periklis Panagopoulos, Efstratios Maltezos, Stylianos Drimis, Angelos Hatzakis

**Affiliations:** 1 Department of Hygiene, Epidemiology and Medical Statistics, Medical School, National and Kapodistrian University of Athens, 75 Mikras Asias Street, 11527 Athens, Greece; ekostakh@med.uoa.gr (E.K.); ahatzak@med.uoa.gr (A.H.); 2 1st Department of Internal Medicine, G. Genimatas GH, 11527 Athens, Greece; p.gargalianos@gna-gennimatas.gr (P.G.); g.xylomenos@gmail.com (G.X.); 3 3rd Internal Medicine Department-Infectious Diseases, Red Cross Hospital, 11526 Athens, Greece; mklazanas@ath.forthnet.gr (M.L.); mariachini@gmail.com (M.C.); 4 5th Department of Medicine and Infectious Diseases, Evaggelismos GH, 10676 Athens, Greece; skoute1@otenet.gr (A.S.); vasileios.papastamopoulos@gmail.com (V.P.); 5 Hellenic Center for Disease Control & Prevention, 15123 Athens, Greece; paraskeva@keelpno.gr; 6 4th Department of Medicine, Attikon GH, Medical School, National and Kapodistrian University of Athens, 12462 Athens, Greece; ananto@med.uoa.gr (A.A.); antpapa1@otenet.gr (A.P.); 7 1st Department of Medicine, Laikon GH, Medical School, National and Kapodistrian University of Athens, 11527 Athens, Greece; mpsichog@yahoo.gr (M.P.); gdaikos@med.uoa.gr (G.L.D.); 8 Department of Internal Medicine, Tzaneio GH, 18536 Piraeus, Greece; gchrysos@gmail.com (G.C.); steliosdrimis@gmail.com (S.D.); 9 HIV/AIDS Unit, A. Syngros Hospital of Dermatology and Venereology, 16121 Athens, Greece; vpaparizos@yahoo.gr (V.P.); kourkounti@in.gr (S.K.); 10 HIV Unit, 2nd Department of Internal Medicine, Hippokration GH, Medical School, National and Kapodistrian University of Athens, 11527 Athens, Greece; helensambatakou@msn.com; 11 Department of Pathophysiology, Laikon GH, Medical School, National and Kapodistrian University of Athens, 11527 Athens, Greece; nsipsas@med.uoa.gr; 12 2nd Department of Internal Medicine, Sismanogleion GH, 15126 Athens, Greece; malvinalada@gmail.com; 13 Department of Internal Medicine, University GH, Democritus University of Thrace, 67100 Alexandroupolis, Greece; ppanago@med.duth.gr (P.P.); emaltez@med.duth.gr (E.M.)

**Keywords:** HIV, molecular epidemiology, drug resistance, transmission dynamics, effective reproductive number

## Abstract

The presence of human immunodeficiency virus type 1 (HIV-1) drug resistance among drug-naïve patients remains stable, although the proportion of patients with virological failure to therapy is decreasing. The dynamics of transmitted resistance among drug-naïve patients remains largely unknown. The prevalence of non-nucleoside reverse transcriptase inhibitors (NNRTI) resistance was 16.9% among treatment-naïve individuals in Greece. We aimed to investigate the transmission dynamics and the effective reproductive number (*R_e_*) of the locally transmitted NNRTI resistance. We analyzed sequences with dominant NNRTI resistance mutations (E138A and K103N) found within monophyletic clusters (local transmission networks (LTNs)) from patients in Greece. For the K103N LTN, the *R_e_* was >1 between 2008 and the first half of 2013. For all E138A LTNs, the *R_e_* was >1 between 1998 and 2015, except the most recent one (E138A_4), where the *R_e_* was >1 between 2006 and 2011 and approximately equal to 1 thereafter. K103N and E138A_4 showed similar characteristics with a more recent origin, higher *R_e_* during the first years of the sub-epidemics, and a declining trend in the number of transmissions during the last two years. In the remaining LTNs the epidemic was still expanding. Our study highlights the added value of molecular epidemiology to public health.

## 1. Introduction

Human immunodeficiency virus type 1 (HIV-1) drug resistance among treatment-naïve patients, hereafter named primary resistance or transmitted resistance, has been shown to compromise first-line response to treatment [1,2,3]. The presence of resistance mutations mostly affects the sensitivity to non-nucleoside reverse transcriptase inhibitors (NNRTIs), due to the low number of mutations (low genetic barrier) required for the development of high levels of resistance to these drugs [4]. The prevalence of primary resistance differed greatly across the globe, where North America (11.5%) and Europe (9.4%) had the highest percentages [5,6]. In all areas, resistance to nucleoside reverse transcriptase inhibitors was the most frequent followed by resistance to NNRTIs [5,6]. However, an increasing trend was found for NNRTI resistance in the Americas/Caribbean, Europe and upper-income Asia. These estimates were modeled by sample year or by year due to antiretroviral treatment scale-up in different regions [5,6,7,8], suggesting that the incidence of resistance is unknown. Estimates of primary resistance have been based mostly on the proportions of newly diagnosed individuals carrying resistance strains [5,6,9], and in a few studies on proportions of resistant strains among newly or acutely infected subjects [10,11,12,13,14,15]. The incidence of primary resistance, therefore, remains largely unknown. 

The overall prevalence of mutations associated with resistance in treatment-naïve individuals was 22.2% for protease (PR) and reverse transcriptase (RT) sequences sampled during 1 January 2003–30 June 2015 in Greece [16]. Resistance to NNRTIs was the most prevalent (16.9%), and our previous analysis revealed that the majority (89%) of NNRTI-resistant viruses (E138A, K103N, and V179D) for subtype A1 belonged to monophyletic clusters (local transmission networks (LTNs)). Specifically, 85.7% of sequences with K103N, and 82.7% with E138A belonged to one and four LTNs, respectively, suggesting that the viruses with the most prevalent NNRTI resistance mutations spread as a result of onward transmission [16]. For subtype B, either non-clustered sequences or small LTNs (two to six sequences) were identified, suggesting limited onward transmission.

In the current analysis, our objective was to infer the temporal characteristics and transmission dynamics for subtype A1 locally transmitted NNRTI-resistant strains by means of phylodynamic analysis. Specifically, we aimed to identify the time of the origin, the number of transmissions (lineages) that is a proxy of the cumulative incidence, and the effective reproductive number (*R_e_*), over time of the locally transmitted resistant strains. 

## 2. Materials and Methods

HIV-1 sequences were available in PR/RT, generated as part of the routine HIV-1 drug resistance testing in Greece [16]. The study population corresponded to patients for whom viral sequences carried the most prevalent NNRTI resistance mutations (E138A and K103N) and were found to belong within monophyletic clusters (LTNs) as previously described [16]. The study was approved by the ethical committee of the Medical School, National and Kapodistrian University of Athens.

Phylodynamic analysis was performed for subtype A1 major LTNs, which consisted of more than six sequences (*N* = 5). Specifically, we analyzed four LTNs including sequences with E138A (*N* = 22, 38, 50 and 38 for clusters 1, 2, 3, and 4, respectively) and one with K103N (*N* = 48) (Table 1). LTNs were defined as follows: (i) phylogenetic clusters including ≥2 sequences from the same geographic area (Greece) at a proportion higher than 75% (geographic criterion), and (ii) highly supported clusters (phylogenetic criterion; Shimodaira-Hasegawa values >0.90) [16,17]. We performed a two-step analysis by using a Bayesian approach: (i) we analyzed the datasets by using the birth-death basic reproductive number (*R*_0_) models as implemented in BEAST v1.8.0 [18], and (ii) we repeated the analyses by using the birth-death skyline serial models as implemented in BEAST v2.1.3 [19,20]. Due to the high complexity of the latter models, convergence of Markov chain Monte Carlo (MCMC) cannot be easily reached. For this reason, we used informative priors for some of the models’ parameters, as explained thoroughly below in the text. Birth-death models (BDM) allow estimation of important epidemiological parameters such as the *R_e_*. *R_e_* is defined as a type of *R*_0_ without the assumption of the totally susceptible population, and thus is defined as the number of expected secondary infections per infected individual at any time point of the epidemic.

In the first step, analysis for each LTN was performed by using the Hasegawa, Kishino, and Yano (HKY + G) as a nucleotide substitution model, and the birth-death *R*_0_ models as implemented in BEAST v1.8.0 [18]. Non-informative priors were used for the MCMC runs. Molecular clock analysis was conducted using the uncorrelated log-normal relaxed molecular clock option. The MCMC analysis comprised 30 × 10^6^ generations, sampled every 3000 steps. The first 3 × 10^6^ generations (10%) were discarded as burn-in. MCMC convergence was investigated by determining whether the effective sample size (ESS) values for each parameter were >100. The resulting log files were visualized in the program Tracer v1.5. The root-to-tip genetic distance against sampling time was plotted using the program TempEst v1.5.1. 

In the second step of the analysis, we used the previously estimated mean clock rate assuming a Normal distribution as informative prior. We also fixed the HKY transitions/transversions ratio and the time of infection of the first person in each LTN (origin) by using the previously estimated values. For the *R*_0_ and the becoming-noninfectious rate, we assumed a LogNormal (0, 10.0), and for the sampling probability a Beta (0, 1.0) prior distribution, respectively. We allowed the becoming-noninfectious rate and the sampling probability parameters to be estimated over time. All the other parameters were defined as previously described at the first step of the analysis. MCMC was run for 30 × 10^6^ generations, sampled every 3000 steps (10% was discarded as burn-in). MCMC convergence was analyzed in the program Tracer v1.5 to ensure that the ESS of all parameters was >100. Each run consensus tree was implied and the maximum clade credibility tree was then found using the program TreeAnnotator v2.1.3 [18]. Graphical representation of the *R_e_* over time was plotted using R.

Demographic data were summarized using absolute and relative frequencies. The statistical analysis was based on Pearson’s chi-square test or Fisher’s exact test, and the correlation between root-to-tip and sampling time was assessed by Spearman’s correlation coefficient in STATA 12-StataCorp LP.

## 3. Results

Our study included subtype A1 sequences with the dominant NNRTI-resistant mutations (E138A and K103N) found to spread within major LTNs in Greece [16]; a clustering pattern driven by onward transmissions of NNRTI-resistant viruses. Our population was drawn from 3,428 treatment-naïve individuals sampled in Greece during the period of 1 January 2003–30 June 2015, corresponding to 39.4% of the total HIV diagnoses (*N* = 8694) reported in the national surveillance system at the Hellenic Center for Disease Control and Prevention in Greece. The prevalence of mutations associated with resistance as estimated using the HIVdb resistance interpretation algorithm was 22.2%. Resistance to NNRTIs was the most prevalent (16.9%), and specifically E138A (7.7%), E138Q (4.0%), and K103N (2.3%) were the most common mutations [16]. The majority of individuals infected with subtype A1 NNRTI-resistant strains (245 out of 273, 89.7%) fell within well-supported monophyletic clusters (LTNs) [16]. We herein estimated the phylodynamic characteristics of NNRTI-transmitted resistance, named as the temporal origin, the number of transmissions (lineages), and the *R_e_*, over time.

Plotting the root-to-tip genetic distance against sampling time revealed significant molecular clock signals in LTNs E138A_2 (*R* = 0.39, *p* = 0.008), E138A_4 (*R* = 0.43, *p* = 0.008), and K103N (*R* = 0.38, *p* = 0.009). For clusters E138A_1 and E138A_3, we found no significant signal, probably due to improper rooting. After the inclusion of a few subtype A1 sequences from the Greek epidemic sampled during 2002–2003 as references, the molecular clock signal was significant in both clusters: E138A_1 (*R* = 0.48, *p* = 0.014) and E138A_3 (*R* = 0.28, *p* = 0.037). 

**Figure 1:**
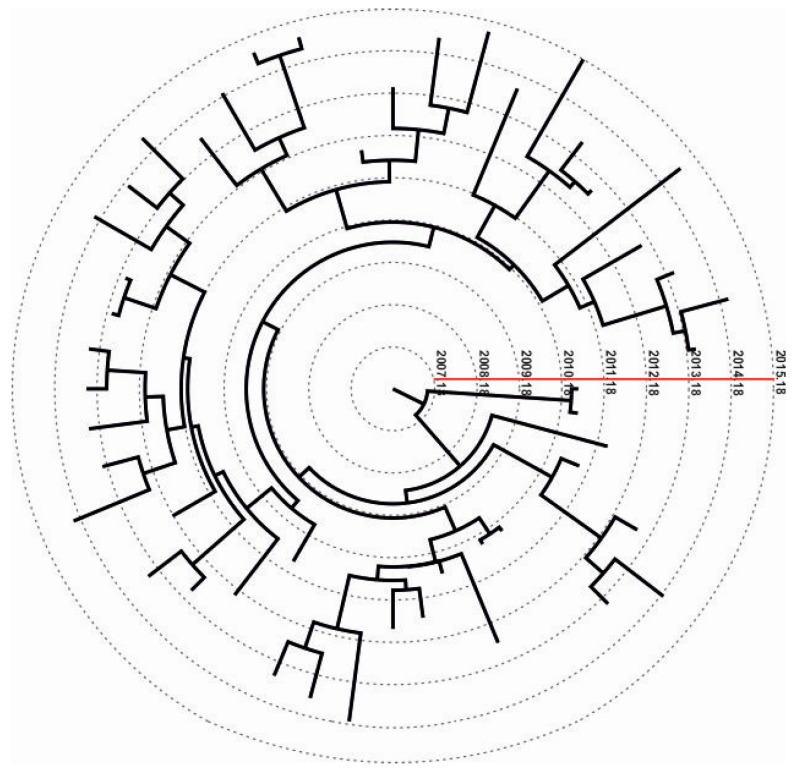
Example of a dated phylogeny (K103N) for one of the five major local transmission networks (LTNs) with resistance. The red line indicates years and the root corresponds to the origin of the K103N sub-epidemic. Dotted lines indicate middle time points across the tree.

Molecular clock analyses revealed that the time of the most recent common ancestor (t_MRCA_) was in 2007 (95% highest posterior density (HPD): 2004–2009) for the K103N LTN (Table 1, Figure 1) versus 1995 (95% HPD: 1991–1999), 1996 (95% HPD: 1989–2000), 1997 (95% HPD: 1991–2001), and 2004 (95% HPD: 2000–2007) for E138A LTNs (Table 1). The t_MRCA_ is considered as the approximate time of infection of the potential founder of the NNRTI-resistant sub-epidemics sampled in our data. These findings suggest that three out of four E138A sub-epidemics originated around the same time point (starting between 1995 and 1997) several years ago (Table 1). In contrast to E138A, the origin of K103N and E138A_4 sub-epidemics was estimated to be more recent (2007 and 2004, respectively) (Table 1). 

**Figure 2:**
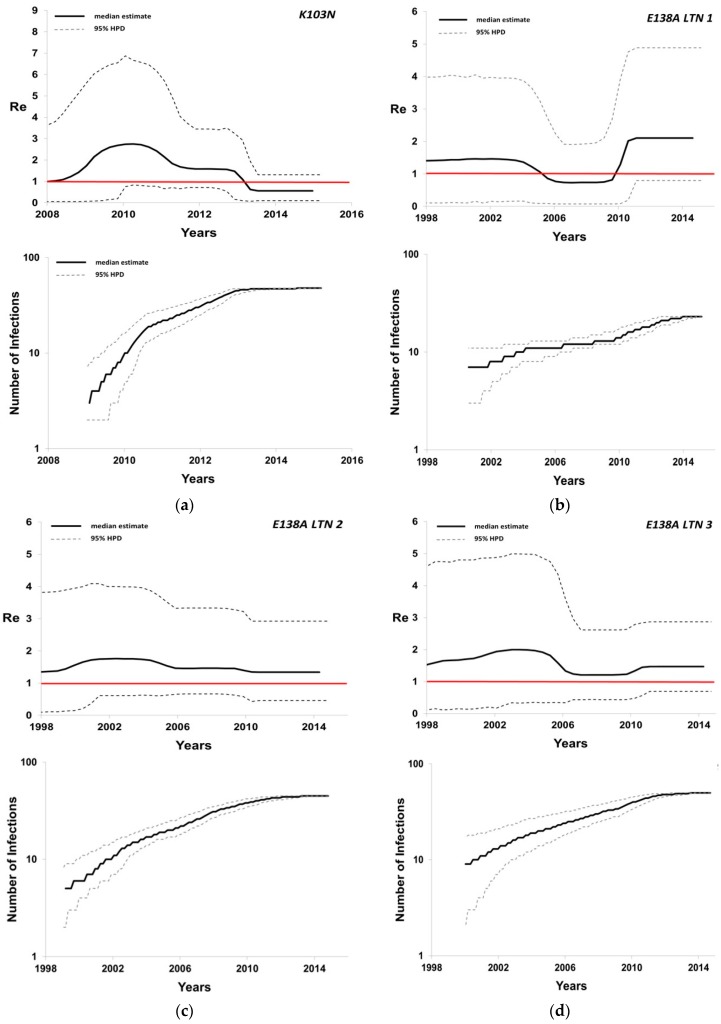

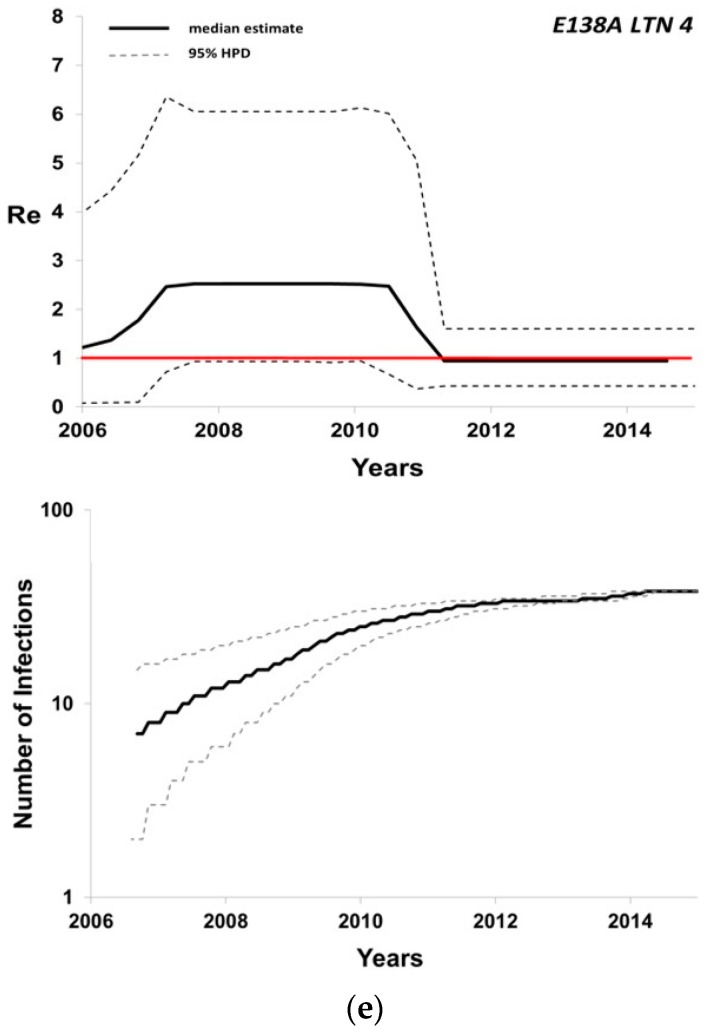
Bayesian skyline plots estimated by BEAST v2.1.3 using birth-death skyline serial models presenting the effective reproductive number (*R_e_*) over time, and the cumulative number of lineages (infections) in a logarithmic scale over time for the five subtype A1 local transmission networks (LTNs) with resistance: (**a**) K103N; (**b**) E138A LTN 1; (**c**) E138A LTN 2; (**d**) E138A LTN 3; (**e**) E138A LTN 4.

The estimated birth-death skyline plots showed significant differences among E138A and K103N LTNs (sub-epidemics) (Table 1, Figure 2a–e). Specifically, the slope of the number of lineages (transmissions) over time estimated at the exponential phase of the BDM skylines for E138A sequences was lower (slope = 0.9, 3.2, 3.1 and 5.5) than that for K103N (slope = 10.5). Similarly, the highest *R_e_* was found for K103N (maximum value of median *R_e_* = 2.8) and the most recent E138A LTN (maximum value of median *R_e_* = 2.5) (E138A_4, Table 1). For the remaining three E138A LTNs, *R_e_* was 1.8, 2.0, and 2.1 (maximum values of median *R_e_*) (Τable 1). Notably, for the K103N LTN, *R_e_* (median estimate) was higher than 1 over a period of almost six years (between 2008 and the first half of 2013), and it started declining in the second half of 2013 (Figure 2a). For E138A LTNs, the *R_e_* (median estimates) was higher than 1 for longer time periods (1998–2015) (Figure 2b–d), except the most recent one where the *R_e_* was higher than 1 between 2006 and 2011 and approximately equal to 1 thereafter (Figure 2e), and the E138A_1 for which the *R_e_* (median estimate) was lower than 1 between 2005 and 2010. The uncertainty, however, on the *R_e_* estimates was large. Although for all E138A LTNs the time of origin was estimated to be several years ago, the number of lineages (transmissions) over time increased in the last few years (2011–2015) (Figure 2b–d), except for E138A_4 that remained in an endemic situation (*R_e_* ~ 1) during this period (Figure 2e). Overall, K103N and E138A_4 LTNs showed similar characteristics, including a recent t_MRCA_, the highest slopes and *R_e_*, as well as a declining trend in the number of transmissions during the last two years of the study period. The remaining E138A LTNs showed an approximately steadily increasing epidemic phase lasting for longer time periods.

To investigate potential differences among the LTNs, we compared populations’ characteristics (i) among the five LTNs, and (ii) between K103N and E138A_4, and E138A_1, E138A_2, and E138A_3 LTNs (Table 2, Supplementary Materials Table S1). The latter grouping was made since K103N and E138A_4 had similar characteristics in their transmission dynamics and a more recent t_MRCA_ versus the others. We found that the distribution of risk groups was different in both comparisons (Table 2, Supplementary Materials Table S1); men who have sex with men (MSM) were at higher proportion in K103N and E138A_4 (77.9%) versus 69.1% for the others (Supplementary Materials Table S1; *p* < 0.05). For E138A_1, E138A_2, and E138A_3, we found a higher proportion of men who have sex with women (11.8%) (Supplementary Materials Table S1). Significant differences were found also for the distribution of nationalities in both groupings (Table 2); however, given the high proportion of unknowns, no conclusions can be drawn about this characteristic. 

## 4. Discussion

In the current study, we estimated the temporal origin and phylodynamic characteristics of the subtype A1 NNRTI-resistant viruses spread to LTNs from treatment-naïve individuals in Greece. We focused on subtype A1, since dominant resistant viruses spreading to major LTNs (*N* > 6 individuals) belonged only to this subtype, except from the E138Q LTN found among people who inject drugs as analyzed previously [21]. 

For subtype A1, E138A LTNs—except that from the E138A_4—were founded more than 15 years ago in contrast to the more recent K103N LTN, suggesting that the E138A resistance network expanded many years before the use of rilpivirine in clinical practice [4]. These findings suggest that resistance mutations with low fitness cost such as E138A can be propagated in the absence of drug-selective pressure [22] as a result of onward transmissions among treatment-naïve individuals. 

Transmission dynamics showed that the more recent LTNs (K103N and E138A_4) initially expanded at higher rates (higher slope and *R_e_*) than those with earlier t_MRCA_. These differences could be due to the higher risk behavior of the individuals infected with K103N and E138A_4, compared to those found within the E138A LTNs 1, 2, and 3. Our analysis suggested that MSM were represented at higher proportions in the former LTNs; however, due to missing data about our populations’ risk behavior, no final conclusion can be made regarding whether any specific behavior was associated with a more rapid transmission of the two LTNs versus the others. Notably, with regard to *R_e_* trends, we found the opposite, where the more recent LTNs (K103N & E138A_4) were declining or remained stable over the last few years (*R_e_* < 1, or *R_e_* = 1), versus E138A LTNs 1, 2, and 3, which continue to expand (*R_e_* > 1). The latter sub-epidemics showed a higher potential than the more recent ones and continue to expand even though they had originated more than 15 years ago. Therefore, knowledge about the time of the origin (i.e. the t_MRCA_) of an epidemic is not necessarily adequate to understand its transmission potential. Evidence about the recent activity of LTNs is important in order to prioritize HIV prevention. For example, although early combination antiretroviral therapy (cART) is recommended for all individuals [23], knowledge about which LTNs remain active can enhance the effectiveness of interventions besides early cART initiation, targeting HIV-infected individuals and their partners. The *R_e_* can provide a useful metric with regard to the transmission dynamics of the LTNs, since *R_e_* > 1 indicates an increasing epidemic, while *R_e_* < 1 denotes a declining phase [20]. 

Previous studies have shown that the global prevalence of NNRTI resistance has been increasing [5,6,9]. In a systematic review of all HIV-1 RT sequences published between 2000 and 2013, it was reported that the prevalence of NNRTI resistance among treatment-naïve individuals has been increasingly detected in the Americas/Caribbean, Europe, and upper-income Asia, while no increasing trend was found for South/Southeast Asia [5,6]. These estimates were mostly based on the year of sampling and, in order to estimate the levels of resistance among recently infected individuals, Rhee et al. performed a nested analysis using only sequences with mixtures at <5% [5,6]. This approach can provide evidence about the incidence of resistance that for most areas is largely unknown. In our study, we show that phylodynamic analysis can be used to estimate transmission dynamics and the number of lineages (transmissions) over time that is a proxy of cumulative incidence. Therefore, molecular epidemiology methods can provide some clues about the trends of transmitted resistance. 

Our study has some limitations; for example, the sampling coverage for the HIV-1 infected population carrying resistant strains clustered within the LTNs is not ideal. For our study population, the sampling coverage was approximately 40%. Moreover, the HPD intervals can be wider for the BDM skyline than the coalescence skyline. In our study, the HPDs were wide for the *R_e_* estimations and for this reason we interpreted the previous in conjunction with the BDM skylines. 

## 5. Conclusions

Previous analyses have also shown that in some cases resistance among treatment-naïve patients was due to onward transmissions [13,24,25,26,27,28]. We herein analyzed the LTNs of major NNRTI resistance mutations to estimate the number of transmissions (lineages) over time and the *R_e_*, thus providing a more detailed picture of the characteristics of resistance sub-epidemics. To the best of our knowledge, this is one of the few studies using molecular epidemiology of HIV-1-resistant viruses to address this issue. The information estimated in the current analysis can be useful to identify trends of transmitted resistance as well as critical epidemiological parameters such as the *R_e_*. This knowledge can be important for public health by identifying priority populations for intervention. Notably, we also show that more recent sub-epidemics do not always continue to expand over earlier ones. Our study highlights the advance of current-state-of-the-art molecular epidemiology methods for the in-depth understanding of epidemics, suggesting that enhanced molecular surveillance can impact HIV prevention.

## Figures and Tables

**Table 1:** Characteristics for the five local transmission networks (LTNs).

Local Transmission Network	Number of Individuals	t_MRCA_^1^ (Median; 95% HPD ^2^)	*R_e_* ^3^ (Maximum Value of Median)	*CV* ^4^ (Median; 95% HPD)
K103N	48	2007 (2004–2009)	2.8	0.25 (0.00002–0.68)
E138A_1	22	1995 (1991–1999)	2.1	1.10 (0.57–1.72)
E138A_2	38	1996 (1989–2000)	1.8	0.73 (0.47–1.04)
E138A_3	50	1997 (1991–2001)	2.0	0.84 (0.48–1.24)
E138A_4	38	2004 (2000–2007)	2.5	0.58 (0.20–0.95)

^1^ t_MRCA_: time of the Most Recent Common Ancestor; ^2^ HPD: Highest Posterior Density; ^3^ *R_e_*: effective reproductive number; ^4^ *CV*: coefficient of variation.

**Table 2:** Characteristics of the study population infected with subtype A1 non-nucleoside reverse transcriptase inhibitors (NNRTI)-resistant viruses spread to local transmission networks (LTNs).

Local Transmission Networks						
	K103N (*N*, %)	E138A_1 (*N*, %)	E138A_2 (*N*, %)	E138A_3 (*N*, %)	E138A_4 (*N*, %)	*p*-value
**Number of individuals**	48 (24.5)	22 (11.2)	38 (19.4)	50 (25.5)	38 (19.4)	
**Gender**						0.159
Male	48 (100.0)	21 (95.5)	36 (94.7)	50 (100.0)	37 (97.4)	
Female	0 (0.0)	1 (4.5)	2 (5.3)	0 (0.0)	1 (2.6)	
**Risk group**						0.046
MSM ^1^	35 (72.9)	13 (59.1)	24 (63.2)	39 (78.0)	32 (84.2)	
PWID ^2^	1 (2.1)	0 (0.0)	1 (2.6)	3 (6.0)	1 (2.6)	
MSW ^3^	1 (2.1)	5 (22.7)	5 (13.2)	3 (6.0)	0 (0.0)	
Unknown	11 (22.9)	4 (18.2)	8 (21.0)	5 (10.0)	5 (13.2)	
**Nationality**						0.020
Hellenic	21 (43.8)	12 (54.6)	27 (71.1)	37 (74.0)	21 (55.3)	
Non Hellenic	2 (4.2)	0 (0.0)	3 (7.9)	1 (2.0)	1 (2.6)	
Unknown	25 (52.0)	10 (45.4)	8 (21.0)	12 (24.0)	16 (42.1)	

^1^ MSM: Men who have Sex with Men; ^2^ PWID: People Who Inject Drugs; ^3^ MSW: Men who have Sex with Women.

## Supplementary Materials

The following are available online at www.mdpi.com/2073-4425/8/11/322/s1. Table S1: Characteristics of the study population grouped according to the phylodynamic characteristics of the local transmission networks (LTNs).

## References

[1] Cozzi-Lepri A., Noguera-Julian M., Di Giallonardo F., Schuurman R., Daumer M., Aitken S., Ceccherini-Silberstein F., D’Arminio Monforte A., Geretti A.M., Booth C.L. (2015). Low-frequency drug-resistant HIV-1 and risk of virological failure to first-line NNRTI-based art: A multicohort european case-control study using centralized ultrasensitive 454 pyrosequencing. J. Antimicrob. Chemother..

[2] Li J.Z., Paredes R., Ribaudo H.J., Svarovskaia E.S., Metzner K.J., Kozal M.J., Hullsiek K.H., Balduin M., Jakobsen M.R., Geretti A.M. (2011). Low-frequency HIV-1 drug resistance mutations and risk of nnrti-based antiretroviral treatment failure: A systematic review and pooled analysis. JAMA.

[3] Wittkop L., Gunthard H.F., de Wolf F., Dunn D., Cozzi-Lepri A., de Luca A., Kucherer C., Obel N., von Wyl V., Masquelier B. (2011). Effect of transmitted drug resistance on virological and immunological response to initial combination antiretroviral therapy for HIV (eurocoord-chain joint project): A european multicohort study. Lancet Infect. Dis..

[4] Wensing A.M., Calvez V., Gunthard H.F., Johnson V.A., Paredes R., Pillay D., Shafer R.W., Richman D.D. (2017). 2017 update of the drug resistance mutations in HIV-1. Top. Antivir. Med..

[5] Rhee S.Y., Blanco J.L., Jordan M.R., Taylor J., Lemey P., Varghese V., Hamers R.L., Bertagnolio S., de Wit T.F., Aghokeng A.F. (2015). Correction: Geographic and temporal trends in the molecular epidemiology and genetic mechanisms of transmitted HIV-1 drug resistance: An individual-patient- and sequence-level meta-analysis. PLoS Med..

[6] Rhee S.Y., Blanco J.L., Jordan M.R., Taylor J., Lemey P., Varghese V., Hamers R.L., Bertagnolio S., Rinke de Wit T.F., Aghokeng A.F. (2015). Geographic and temporal trends in the molecular epidemiology and genetic mechanisms of transmitted HIV-1 drug resistance: An individual-patient- and sequence-level meta-analysis. PLoS Med..

[7] Balode D., Westman M., Kolupajeva T., Rozentale B., Albert J. (2010). Low prevalence of transmitted drug resistance among newly diagnosed HIV-1 patients in Latvia. J. Med. Virol..

[8] SPREAD programme (2008). Transmission of drug-resistant HIV-1 in Europe remains limited to single classes. AIDS.

[9] Hofstra L.M., Sauvageot N., Albert J., Alexiev I., Garcia F., Struck D., Van de Vijver D.A., Asjo B., Beshkov D., Coughlan S. (2016). Transmission of HIV drug resistance and the predicted effect on current first-line regimens in Europe. Clin. Infect. Dis..

[10] Wensing A.M., van de Vijver D.A., Angarano G., Asjo B., Balotta C., Boeri E., Camacho R., Chaix M.L., Costagliola D., De Luca A. (2005). Prevalence of drug-resistant HIV-1 variants in untreated individuals in Europe: Implications for clinical management. J. Infect. Dis..

[11] Castor D., Low A., Evering T., Karmon S., Davis B., Figueroa A., LaMar M., Garmon D., Mehandru S., Markowitz M. (2012). Transmitted drug resistance and phylogenetic relationships among acute and early HIV-1-infected individuals in New York city. J. Acquir. Immune Defic. Syndr..

[12] Boden D., Hurley A., Zhang L., Cao Y., Guo Y., Jones E., Tsay J., Ip J., Farthing C., Limoli K. (1999). HIV-1 drug resistance in newly infected individuals. JAMA.

[13] Brenner B.G., Roger M., Moisi D.D., Oliveira M., Hardy I., Turgel R., Charest H., Routy J.P., Wainberg M.A. (2008). Transmission networks of drug resistance acquired in primary/early stage HIV infection. AIDS.

[14] Toni T.A., Asahchop E.L., Moisi D., Ntemgwa M., Oliveira M., Masquelier B., Brenner B.G., Wainberg M.A. (2009). Detection of human immunodeficiency virus (HIV) type 1 M184V and K103N minority variants in patients with primary HIV infection. Antimicrob. Agents Chemother..

[15] Brenner B.G., Roger M., Routy J.P., Moisi D., Ntemgwa M., Matte C., Baril J.G., Thomas R., Rouleau D., Bruneau J. (2007). High rates of forward transmission events after acute/early HIV-1 infection. J. Infect. Dis..

[16] Paraskevis D., Kostaki E., Magiorkinis G., Gargalianos P., Xylomenos G., Magiorkinis E., Lazanas M., Chini M., Nikolopoulos G., Skoutelis A. (2017). Prevalence of drug resistance among HIV-1 treatment-naïve patients in Greece during 2003–2015: Transmitted drug resistance is due to onward transmissions. Infect. Genet. Evol..

[17] Paraskevis D., Nikolopoulos G., Tsiara C., Paraskeva D., Antoniadou A., Lazanas M., Gargalianos P., Psychogiou M., Malliori M., Kremastinou J. (2011). HIV-1 outbreak among injecting drug users in Greece, 2011: A preliminary report. Euro Surveill..

[18] Drummond A.J., Suchard M.A., Xie D., Rambaut A. (2012). Bayesian phylogenetics with BEAUti and the BEAST 1.7. Mol. Biol. Evol..

[19] Bouckaert R., Heled J., Kuhnert D., Vaughan T., Wu C.H., Xie D., Suchard M.A., Rambaut A., Drummond A.J. (2014). BEAST 2: A software platform for bayesian evolutionary analysis. PLoS Comput. Biol..

[20] Stadler T., Kuhnert D., Bonhoeffer S., Drummond A.J. (2013). Birth-death skyline plot reveals temporal changes of epidemic spread in HIV and hepatitis C virus (HCV). Proc. Natl. Acad. Sci. USA.

[21] Paraskevis D., Paraschiv S., Sypsa V., Nikolopoulos G., Tsiara C., Magiorkinis G., Psichogiou M., Flampouris A., Mardarescu M., Niculescu I. (2015). Enhanced HIV-1 surveillance using molecular epidemiology to study and monitor HIV-1 outbreaks among intravenous drug users (IDUS) in Athens and Bucharest. Infect. Genet. Evol..

[22] Yang W.L., Kouyos R.D., Boni J., Yerly S., Klimkait T., Aubert V., Scherrer A.U., Shilaih M., Hinkley T., Petropoulos C. (2015). Persistence of transmitted HIV-1 drug resistance mutations associated with fitness costs and viral genetic backgrounds. PLoS Pathog..

[23] European AIDS Clinical Society (2017). Guidelines.

[24] Antoniadou Z.A., Kousiappa I., Skoura L., Pilalas D., Metallidis S., Nicolaidis P., Malisiovas N., Kostrikis L.G. (2014). Short communication: Molecular epidemiology of HIV type 1 infection in northern Greece (2009–2010): Evidence of a transmission cluster of HIV type 1 subtype a1 drug-resistant strains among men who have sex with men. AIDS Res. Hum. Retrovir..

[25] Drescher S.M., von Wyl V., Yang W.L., Boni J., Yerly S., Shah C., Aubert V., Klimkait T., Taffe P., Furrer H. (2014). Treatment-naive individuals are the major source of transmitted HIV-1 drug resistance in men who have sex with men in the swiss HIV cohort study. Clin. Infect. Dis..

[26] Mbisa J.L., Fearnhill E., Dunn D.T., Pillay D., Asboe D., Cane P.A. (2015). Evidence of self-sustaining drug resistant HIV-1 lineages among untreated patients in the United Kingdom. Clin. Infect. Dis..

[27] Mourad R., Chevennet F., Dunn D.T., Fearnhill E., Delpech V., Asboe D., Gascuel O., Hue S. (2015). A phylotype-based analysis highlights the role of drug-naive HIV-positive individuals in the transmission of antiretroviral resistance in the UK. AIDS.

[28] Theys K., Van Laethem K., Gomes P., Baele G., Pineda-Pena A.C., Vandamme A.M., Camacho R.J., Abecasis A.B. (2016). Sub-epidemics explain localized high prevalence of reduced susceptibility to rilpivirine in treatment-naive HIV-1-infected patients: Subtype and geographic compartmentalization of baseline resistance mutations. AIDS Res. Hum. Retrovir..

