# Supplementary material for: Transmission Dynamics of HIV-1 Drug Resistance among Treatment-Naïve Individuals in Greece: The Added Value of Molecular Epidemiology to Public Health
Source: Genes (Basel). 2017 Nov 13;8(11):322. doi: 10.3390/genes8110322 (PMC5704235; doi:10.3390/genes8110322)
Supplement: Supplementary file 1 [file genes-08-00322-s001.pdf]

Supplementary Table 1: Characteristics of the study population grouped according to the phylodynamic characteristics of the local transmission networks (LTNs)

	LTNs		
	**K103N & E138A_4 (N, %)**	**E138A_1, E138A_2 & E138A_3 (N, %)**	***p*-value**
**Number of individuals**	86 (43.9)	110 (56.1)	
**Gender**			0.633
Male	85 (98.8)	107 (97.3)	
Female	1 (1.2)	3 (2.7)	
**Risk group**			0.020
MSM[1]	67 (77.9)	76 (69.1)	
PWID[2]	2 (2.3)	4 (3.6)	
MSW[3]	1 (1.2)	13 (11.8)	
Unknown	16 (18.6)	17 (15.5)	
**Nationality**			0.011
Hellenic	42 (48.8)	76 (69.1)	
Non Hellenic	3 (3.5)	4 (3.6)	
Unknown	41 (47.7)	30 (27.3)	

[1]MSM: men who have sex with men

[2]PWID: people who inject drugs

[3]MSW: men who have sex with women
